# Frugal Innovation: A Modified Conduit to Enhance the Utility of Semirigid Thoracoscopy

**DOI:** 10.7759/cureus.91489

**Published:** 2025-09-02

**Authors:** Saurabh Karmakar, Shilpi Karmakar

**Affiliations:** 1 Department of Pulmonary Medicine, All India Institute of Medical Sciences, Patna, Patna, IND; 2 Department of Burns and Plastic Surgery, All India Institute of Medical Sciences, Jodhpur, Jodhpur, IND

**Keywords:** frugal innovation, ideal conduit, innovation, pleural fluid (pf), semirigid thoracoscopy

## Abstract

The landscape of modern healthcare is becoming increasingly complex, with rising costs of diagnostic and therapeutic interventions. In low- and middle-income countries, such as India, affordability remains a major barrier to accessing advanced medical care. The All India Institute of Medical Sciences (AIIMS), Patna, India, was established with the aim of providing high-quality care at minimal cost, ensuring access even for the most underserved patients. Medical thoracoscopy is an invaluable diagnostic and therapeutic procedure for pleural diseases. Semirigid thoracoscopes, such as the Olympus EVIS EXERA LTF-160 (Olympus America, Center Valley, PA, USA), allow direct visualization and biopsy of pleural pathology under local anesthesia. However, their use is restricted in resource-limited settings due to the high cost of proprietary accessories, specifically the single-use trocar and cannula systems (e.g., Olympus MAJ-1058, Olympus Medical Systems Corp., Tokyo, Japan), which are not economically viable for repeated use in underfunded public healthcare systems. To address this challenge, we developed a frugal and functional innovation to replace the proprietary trocar-cannula system. This system integrates a reusable surgical accessory with a nonreusable medical device to substitute for the costly, manufacturer-provided single-use accessory. This report presents the technique, cost-benefit analysis, and preliminary safety evaluation of this innovation in clinical use.

## Introduction

The semirigid thoracoscope is the modality of choice for investigating pleural effusion of unknown etiology [1]. It is introduced into the thoracic cavity through a flexible plastic trocar-cannula assembly, which is a single-use disposable device. The use of disposable components increases the cost of the procedure for patients and contributes to the overall expenses of solid waste disposal and consumables. Additionally, reliance on single-use components creates dependence on suppliers and the supply chain.

We describe a new innovation designed as a thoracoscope conduit to substitute for the manufacturer-specified trocar and cannula. Our modified thoracoscope conduit utilizes locally available consumables, thereby drastically reducing the cost of disposable accessories. Medical thoracoscopy (MT) will continue to advance with the wider adoption of such innovations in equipment, enabling a broader range of interventional procedures.

## Technical report

**Figure 1:**
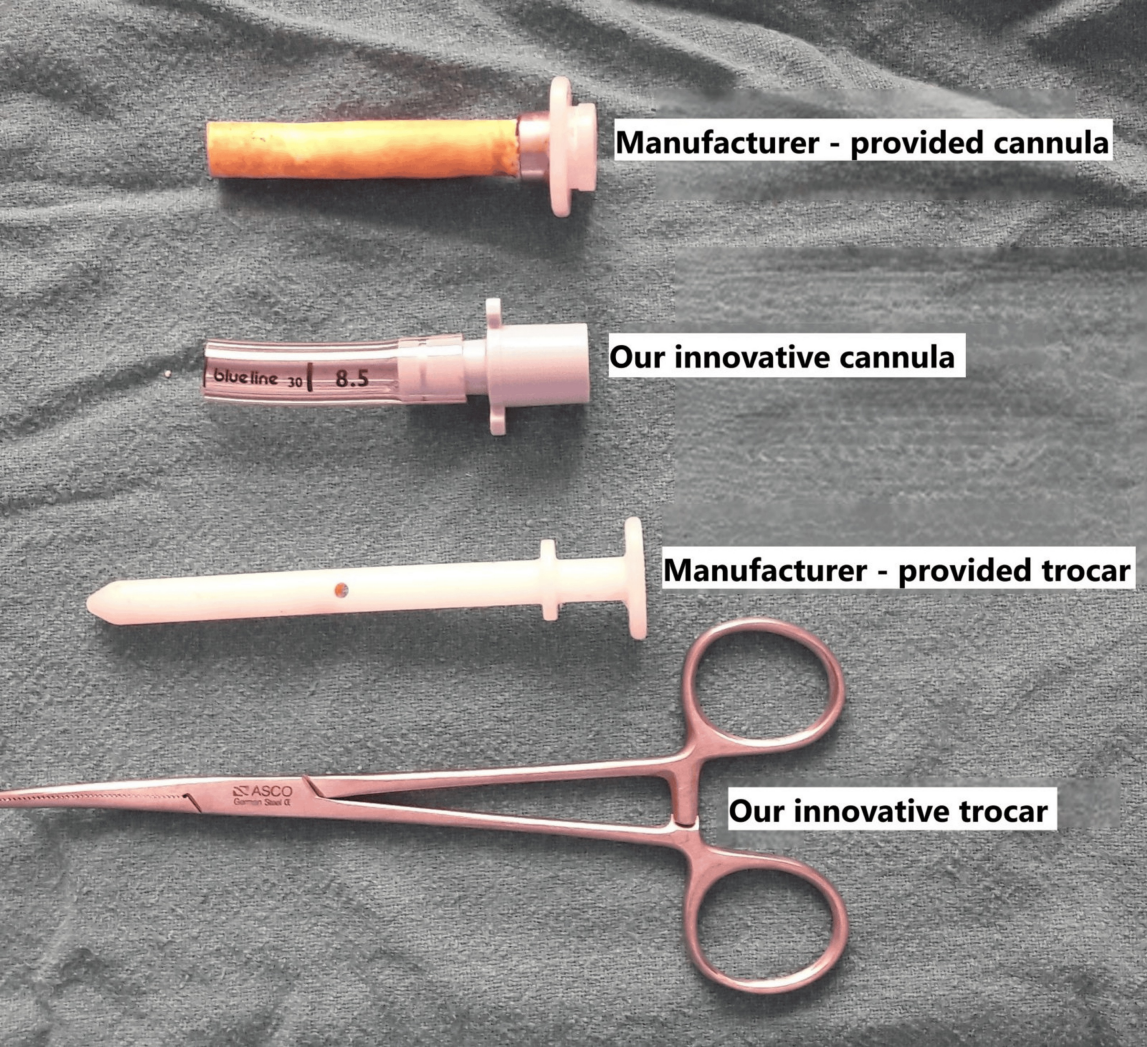
Component parts of the manufacturer-provided trocar-cannula and the innovative trocar-cannula The cut ET tube closely resembles the manufacturer-provided cannula, while the curved artery forceps replicates the functionality of the manufacturer-provided trocar. ET, endotracheal

**Figure 2:**
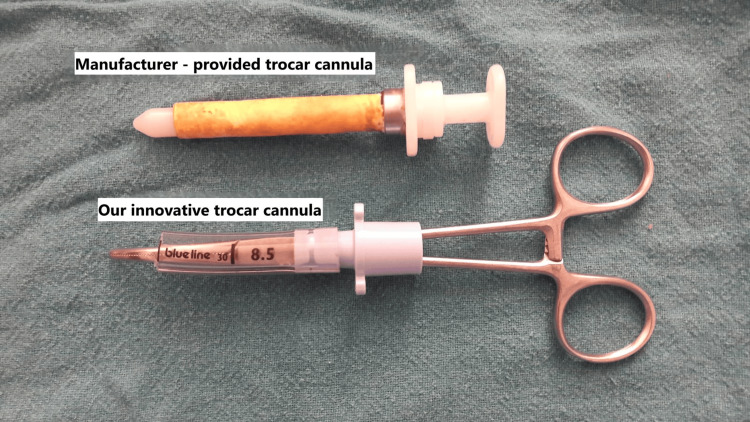
Manufacturer-provided trocar-cannula and the innovative trocar-cannula after assembly The resemblance between the two systems is striking, although their costs differ substantially.

Our department uses the semirigid thoracoscope EVIS EXERA LTF-160 (Olympus America, Center Valley, PA, USA) for MT in approximately 10 patients with undiagnosed pleural effusion each week. The MAJ-1058 8 mm FLEX TROCAR (Olympus Medical Systems Corp., Tokyo, Japan) is employed as the standard trocar-cannula system to access the pleural space. All procedures are performed in accordance with the British Thoracic Society guidelines for thoracoscopy [1]. Following patient preparation and the initial steps of semirigid thoracoscopy, the MAJ-1058 trocar-cannula assembly is inserted through the tract into the pleural space. The trocar is then removed, and the thoracoscope is advanced through the cannula. Our innovation replaces the proprietary system with two readily available components: a 6-inch curved steel artery forceps, used as the trocar, and a PORTEX endotracheal (ET) tube, size 8.5 (outer diameter 11.6 mm; Smiths Medical, Inc., Minneapolis, MN, USA), cut to 7.4 cm in length from the proximal end, used as the cannula (Figure 1, Figure 2).

**Figure 3:**
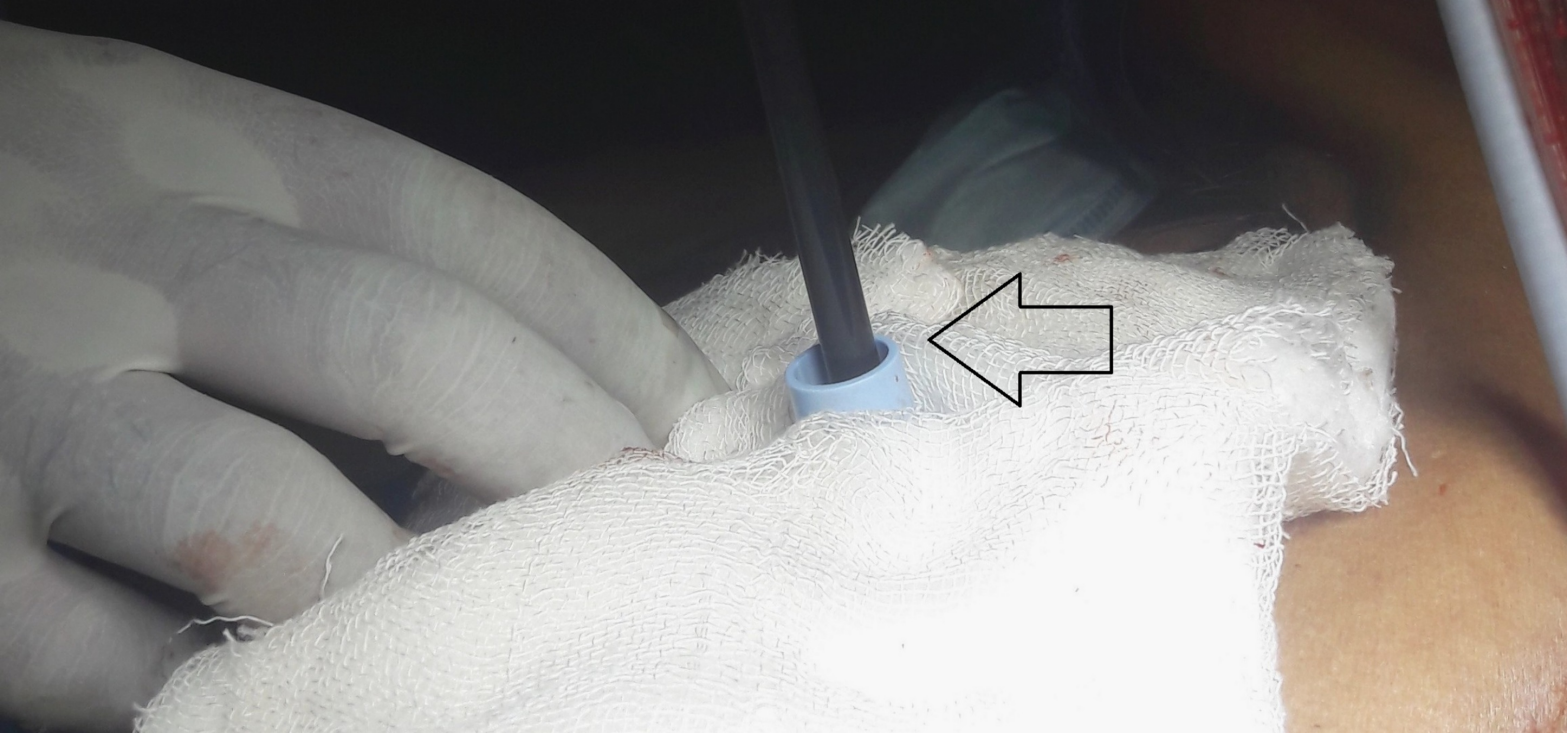
Semirigid thoracoscopy performed using the innovative cannula After the assembly was introduced into the tract and the trocar (curved artery forceps) was withdrawn, the thoracoscope was advanced through the innovative cannula. No perceptible difference was observed compared with passage through the manufacturer-provided trocar-cannula system.

In our thoracoscopy conduit innovation, the connector of the ET tube serves as the proximal end, while the cut end functions as the distal end. The curved artery forceps is inserted into the shortened ET tube, and the assembly is held between the thumb and index finger before being gently advanced into the tract with a downward motion. The curvature of the forceps guides the inward and downward placement of the assembly. Once the conduit is positioned within the tract, the forceps is carefully withdrawn, while the cut ET tube is stabilized by gently pressing its connector downward. The thoracoscope, with an outer diameter of 7 mm, fits snugly into the ET tube (Figure 3).

The MAJ-1058 8MM FLEX TROCAR model trocar and cannula is priced at $100 per box of five. When converted to Indian currency, the cost of a single trocar-cannula is approximately INR 1505. In contrast, our innovation consists of a reusable curved steel artery forceps (INR 145) and a single-use ET tube, size 8.5 (INR 65 per procedure), reducing the recurring cost from INR 1505 to INR 65. This represents a 96% cost savings compared with the manufacturer-approved accessory. In our system, the curved steel artery forceps, owing to its stiffness and wedge-shaped contour, enlarges the tract to the required diameter and facilitates gentle entry into the pleural space along a curved path. The size 8.5 ET tube, made of polyvinyl chloride coated with soft silicone, splints the tract to reduce bleeding, minimizes trauma to surrounding tissues, and provides sufficient rigidity to protect the fiber optics of the thoracoscope body. The curved artery forceps is reusable after autoclaving. We randomized patients undergoing MT over one week in a 1:1 ratio to either our innovative thoracoscopy conduit or the MAJ-1058 8MM FLEX TROCAR system. No variation was observed in post-procedure pain or local complications (wound infection, hematoma, or lung parenchymal injury) between the two trocar-cannula systems.

## Discussion

The rising costs of healthcare pose significant challenges to the accessibility and affordability of advanced medical procedures. This is particularly true in low- and middle-income countries, where healthcare systems must contend with limited resources [2]. Strengthening these systems requires targeted strategies and an environment that fosters innovation [2]. Innovative approaches are needed to deliver high-quality care at reduced costs. This need is especially relevant in tertiary referral centers such as ours, where budgets are constrained but demand for effective medical interventions remains high. Frugal innovation describes the development of solutions designed to serve low-income populations, primarily in developing countries, through affordable products and services that address pressing healthcare problems [3].

Pleural diseases encompass a wide range of disorders associated with substantial morbidity and mortality. The introduction of the semirigid thoracoscope has revolutionized their diagnosis and management, enabling direct visualization and pleural biopsy under local anesthesia [4]. The semirigid thoracoscope (model LTF 160, Olympus Medical Systems Corp.) is ergonomically similar to a fiberoptic bronchoscope, making it well-suited for use by pulmonologists [5]. It is typically introduced into the pleural space through a dedicated single-use disposable trocar and cannula system (MAJ-1058, Olympus Medical Systems Corp.). However, reliance on disposable components increases costs for patients. Moreover, advanced procedural practices and safety protocols encourage greater use of single-use devices, thereby increasing biomedical waste generation [6]. This waste burden is especially concerning in resource-limited settings, where waste management systems may already be inadequate [6]. By incorporating a reusable, autoclave-sterilized component (a curved artery forceps) into our innovation, we were able to reduce costs and minimize the environmental impact of thoracoscopy. The COVID-19 pandemic further highlighted vulnerabilities in global supply chains, including disruptions in the availability of manufacturer-supplied single-use components [7]. During this period, as providers of interventional pulmonology procedures, we faced significant resource constraints. Through frugal innovation, we developed an alternative conduit that substituted expensive proprietary accessories with readily available local materials. Our system employed reusable steel artery forceps combined with a modified single-use ET, reducing procedure costs by 96% compared with the proprietary trocar-cannula. The artery forceps provided stiffness and guided tract patency, while the ET tube acted as a splint to reduce bleeding and tissue trauma. This system was user-friendly, reproducible, and adaptable to other institutions facing similar challenges.

This innovation may be particularly useful in high-volume, economically disadvantaged centers performing multiple thoracoscopies daily. A stock of sterilized artery forceps and disposable ET tubes can be maintained for rapid sequential use, allowing for efficient patient output. Integrating a reusable surgical instrument with a single-use medical device represents a novel strategy to counter the challenges posed by reliance on proprietary disposables [8]. Reusable devices, such as our system, offer improved environmental sustainability compared with disposable counterparts. This model may serve as a blueprint for similar cost-effective adaptations in other medical procedures where high costs and supply chain dependencies hinder access.

Although our preliminary data demonstrated comparable outcomes between the modified thoracoscope conduit and the proprietary system, further validation is warranted. Larger randomized trials, incorporating objective metrics such as procedure duration, operator satisfaction, complication rates, and patient-reported outcomes, will be critical to fully assess safety and efficacy. Rigorous documentation of outcomes and complications will also address potential ethical concerns and strengthen the evidence base supporting our innovation. The history of medical progress is characterized by continuous innovation, and proceduralists in developing countries often must act as innovators themselves. Recognition of such efforts fosters a culture of innovation within healthcare institutions.

In summary, our modified thoracoscope conduit represents a significant advancement for MT in resource-limited settings. By lowering costs and enhancing accessibility, this frugal innovation has the potential to improve patient care and broaden access to advanced procedures. Future studies with larger cohorts and long-term follow-up are essential to validate the technique and support its routine integration into thoracoscopic practice. By achieving substantial cost reduction, focusing on functionality, and maintaining optimal performance, our innovation fulfills the three core criteria of frugal innovation [9]. As healthcare systems continue to evolve, the adoption of frugal innovations will be critical to bridging the gap between advanced medical care and the populations that need it most.

## Conclusions

We present a simple and cost-effective alternative to proprietary trocar-cannula systems for semirigid thoracoscopy. Developed in a tertiary care public institution, this innovation achieves substantial cost reduction without compromising procedural safety or efficacy. Preliminary data indicate comparable outcomes to standard accessories, highlighting its potential to improve access in resource-limited settings. This frugal innovation demonstrates how locally available solutions can address global healthcare challenges. Larger studies with extended follow-up are warranted to validate its effectiveness and support adoption into routine clinical practice.
